# Enhancing uptake of antidiabetic drugs to reduce cardiometabolic risk and dementia burden

**DOI:** 10.1002/alz.70614

**Published:** 2025-09-02

**Authors:** Timothy Daly, Bruno P. Imbimbo

**Affiliations:** ^1^ UMR 1219 Bordeaux Population Health Université de Bordeaux & INSERM Bordeaux France; ^2^ UMR 5164, ImmunoConcept University of Bordeaux & CNRS Bordeaux France; ^3^ Bioethics Program FLACSO Argentina Buenos Aires Argentina; ^4^ Research & Development Chiesi Farmaceutici Parma Italy

1

Cardiometabolic diseases (CMDs) are interrelated conditions that include cardiovascular diseases such as heart attack and stroke, type 2 diabetes, hypertension, dyslipidemia, and obesity that share common risk factors across insulin resistance, chronic inflammation, and lifestyle behaviors associated with ill health, and significantly increase the risk of heart disease and cognitive decline. Metabolic syndrome (MetS) refers to a related cluster of conditions such as abdominal obesity, high blood pressure, elevated blood sugar, high triglycerides, and low high‐density lipoprotein cholesterol that co‐occur and increase the risk of cardiovascular disease, type 2 diabetes, and dementia.

Recently, Karway et al.^1^ conducted a population‐based Medicare analysis of over 20 million individuals to assess the combined effect of CMDs on dementia risk. They found that 37% of dementia cases could be prevented by addressing eight CMDs: diabetes, hyperlipidemia, hypertension, stroke, chronic heart failure, ischemic heart disease, atrial fibrillation, and acute myocardial infarction. We argue that the cardioprotective effects of antidiabetic drugs like glucagon‐like peptide‐1 receptor agonists (GLP‐1RAs), sodium‐glucose cotransporter 2 inhibitors (SGLT2is), and metformin could be leveraged as part of a broader public health strategy to mitigate cardiometabolic risk of dementia.

Growing evidence suggests a synergistic impact of cardiovascular^2^ and metabolic^3^ risk factors across the life course on the development of later life and younger onset dementia.^4^, ^5^ Multiple components of MetS such as hypertension, hyperglycemia, and obesity may contribute to cognitive impairment through various mechanisms. These include cerebral vascular remodeling, chronic cerebral hypoperfusion, impaired insulin signaling in the brain, and activation of neuroinflammatory pathways.^3^ MetS may also exacerbate neurodegeneration, as evidenced by autopsy and neuroimaging studies showing correlations between MetS and widespread structural alterations in the cortex, gray matter, and white matter.^3^

The closer temporal proximity of hypertension, diabetes, and smoking to the clinical onset of dementia, versus more distal risk factors such as physical inactivity or obesity, provides stronger support for a causal relationship. The leading proposed mechanism for these three vascular risk factors is arteriosclerotic cerebral small vessel disease^2^ where the brain's small arteries and arterioles become narrowed and hardened due to arteriosclerosis. Despite the hypothesized importance of this mechanism, specific in vivo biomarkers to measure cerebral small vessel disease such as arteriolosclerosis are only just emerging^6^ against a backdrop of imaging methods that have previously only been able to detect indirect, non‐specific features such as white matter hyperintensities, microbleeds, and enlarged perivascular spaces.

A recent study on the national uptake of cardioprotective antidiabetic drugs between 2015 and 2020 found overall low usage relative to the prevalence of cardiometabolic risk in the population, particularly for people with lower income and less frequent health‐care visits.^7^ Nevertheless, the evidence base for the neuroprotective and cardiometabolic effects of antidiabetic medications requires further strengthening. Methods to achieve this include randomized controlled trials such as the ongoing EVOKE and EVOKE+ studies of semaglutide in Alzheimer's disease,^8^ target trial emulation,^9^ and pragmatic clinical trials.^10^

In conclusion, converging and emerging mechanistic and epidemiological findings suggest that broader access to cardioprotective antidiabetic drugs in the aging population may improve public brain health by targeting cardiometabolic risk factors that contribute to cardiovascular and neurodegenerative diseases. Biomarkers to measure arteriosclerotic cerebral small vessel disease should be further developed within a broader research program to investigate and ultimately mitigate cardiometabolic risk factors for cognitive decline.

## CONFLICT OF INTEREST STATEMENT

Dr. Timothy Daly has no conflicts of interest to declare. Dr. Bruno P. Imbimbo is an employee at Chiesi Farmaceutici. He is listed as an inventor in a number of Chiesi Farmaceutici's patents of anti‐Alzheimer's disease drugs. Author disclosures are available in the supporting information.

## Supporting information



Supporting information
